# Affordance, usefulness, enjoyment, and aesthetics in sustaining virtual reality engagement

**DOI:** 10.1038/s41598-023-42113-1

**Published:** 2023-09-12

**Authors:** Hyeon Jo, Do-Hyung Park

**Affiliations:** 1 HJ Institute of Technology and Management, 71 Jungdong-ro 39, Bucheon-si, Gyeonggi-do 14721 Republic of Korea; 2 Graduate School of Business IT, Kookmin University, 77, Jeongneung-ro, Seongbuk-gu, Seoul, 02707 Republic of Korea

**Keywords:** Psychology, Mathematics and computing

## Abstract

As virtual reality (VR) continues to develop, it's attracting an increasing number of consumers who are seeking more diverse functions and experiences. This study presents a theoretical model designed to identify predictors of VR users' continuance intentions. Data was collected from VR users who had firsthand experiences with the technology, and partial least squares structural equation modeling was employed to analyze this data. The results showed a significant correlation between functional affordance and perceived usefulness. Cognitive affordance was found to have a significant association with perceived usefulness, but it also influenced perceived enjoyment. Moreover, physical affordance significantly related to both perceived usefulness and enjoyment. Perceived usefulness was found to directly affect both attitude and continuance intention, while empirical results validated the impact of perceived enjoyment on attitude. The element of shape showed a significant correlation with attitude. Finally, attitude was found to have a significant association with continuance intention. The findings from this study will provide valuable insights for VR companies, developers, and consumers.

## Introduction

The evolution of technology has introduced humans to an entirely new realm of experiences. Augmented reality (AR) and virtual reality (VR) technologies have surfaced as game-changers in numerous industries, transforming how we interact with digital information and becoming an increasingly integral part of our daily lives. Smartphones have facilitated the use of AR^1^, while many manufacturers are creating VR devices to offer consumers uniquely immersive experiences^2^. VR users don headsets to play games, learn, and perform work-related tasks^3^. Consequently, VR adds value by becoming a part of an expanding spectrum of users’ lives. In 2021, the VR market was valued at USD 21.83 billion^4^, with projections estimating it to reach $20.9 billion at a CAGR of 27.9% by 2025^5^.

VR technologies have gained increasing popularity across various sectors, including education, healthcare, entertainment, and tourism. According to a study by^6^, VR technologies can enhance learning outcomes by facilitating a more engaging and interactive experience. Highlighted that VR technology has been employed to treat various phobias, like acrophobia and aviophobia^7^. Additionally, VR has been utilized to create immersive gaming experiences, thereby offering a more engaging and interactive platform^8^. In light of these advancements, this study aims to identify the key antecedents of continuance intention VR device users.

The drivers of continuance intention for VR users are multifaceted and multidimensional. Cognitive, emotional, and design-related elements play pivotal roles in guiding an individual's decision to persistently engage with VR technology. The concept of affordance, deeply entrenched in Gibson^9^ studies, elucidates the action possibilities offered by a specific environment or object. Within the VR milieu, affordances pertain to the potential actions and functions presented by VR devices^10^. Indeed, products empower users to undertake specific actions through facets like screen design^10,11^. Visceral affordance, behavioral affordance, and reflective affordance influence mobile app user behavior^12^. In the realm of IoT apps, user behavior is stimulated by emotional aspects, tangible elements, and functional determinants^13^. Affordance types such as screen layout, imagery, and icons sway the behaviors of mobile device users^14^. Considering VR users don headsets and interact with expansive screens, it's plausible that these screen elements have a pronounced impact on user behaviors. The affordances of a technology underpin users' descriptive beliefs, which subsequently shape generalized beliefs and ultimately attitudes^15^. With VR being intrinsically interactive, grasping its affordances is key to discerning the motivations propelling users to sustain their engagement.

Defined as the degree to which a person believes that using a particular system would enhance his or her performance, perceived usefulness is a core determinant in technology acceptance model (TAM)^16^. Numerous studies have consistently found perceived usefulness to be a strong predictor of technology adoption and continued use, including in the VR domain^16–21^. Beyond utility, technology usage is also driven by hedonic motivations^22–25^. Perceived enjoyment refers to the intrinsic pleasure one derives from using the technology, without any performance-related outcomes^24^. Given that VR provides immersive experiences, understanding the enjoyment aspect is pivotal. Prior research has shown that perceived enjoyment can be a more influential factor than perceived usefulness in predicting continuance intention, especially for hedonic systems like VR^26^.

The visual and tactile design of VR devices and interfaces, often referred to as aesthetics, plays a pivotal role in the user's overall experience^27^. Consumers tend to favor aesthetically pleasing products^28^. The importance of aesthetics in purchasing decisions has been well-documented in marketing literature^29–32^. Consumers are attracted to more vibrant screen configurations and perceive value in more colorful products^33^. As VR screens strive to mirror reality, they need to be visually appealing. Screens with superior aesthetics are likely to elicit more favorable responses from VR users. The aesthetic appeal of a VR device or interface can influence user satisfaction, which in turn, impacts their continuance intention^34^. In a domain like VR, where the visual experience is central, the influence of aesthetics becomes even more pronounced. In conclusion, a holistic understanding of continuance intention in VR should encompass both utilitarian (affordance and perceived usefulness) and hedonic (perceived enjoyment and aesthetics) factors. These elements collectively provide a comprehensive framework for explaining and predicting VR user behavior over time.

In this study, we aim to delve into the nuanced relationships and interactions in the context of VR user experience. Our specific research questions are:

1. How does functional affordance influence perceived usefulness in a VR context?
2. What roles do cognitive and physical affordances play in shaping both perceived usefulness and perceived enjoyment for VR users?
3. How are perceived usefulness, perceived enjoyment, aesthetics, and shape intricately intertwined to form a user's attitude towards VR?
4. Lastly, how does this formulated attitude, combined with perceived usefulness, drive a user's continuance intention with VR technology?

This study seeks to address the research gap in several ways. First, it introduces the concept of affordance to explain VR usage behavior. While numerous scholars have posited that affordance design plays a crucial role in the use of electronic consumer products^35–37^, research on VR remains limited. This paper contributes to academia by explaining VR user behavior based on affordance design. Second, it considers the aesthetics of the screen to understand users' continuance intention. Given that VR aims to mirror reality, the screen design should feature a splendid color palette and composition. This study is significant as it identifies the impact of aesthetics on users' attitudes. Lastly, this research includes tactile sensation in describing behavioral intention. VR users handle the headsets and wear them on their heads. Some devices even come equipped with haptic functionality^38^. This study adds to the existing literature by revealing the influence of tactile sensation on user behavior.

The remainder of this paper is organized as follows: section “Theoretical background” reviews VR user behavior, affordances, and aesthetics. Section “Research model” presents the research model. Section “Research methodology” describes the measurement tools and samples. Section “Results” outlines the analysis results. Section “Discussion” discusses the findings, and section “Conclusion” concludes the paper.

## Theoretical background

VR technologies have been widely researched in various fields. Researchers have noted that VR technologies hold the potential to revolutionize the way we interact with digital information, offering a more immersive and engaging experience. These technologies can amplify our senses, ushering in new methods for learning, working, and playing. In this section, we'll delve into research studies that have explored user behavior, affordance, and aesthetics in relation to VR.

### VR user behavior

Numerous researchers have probed the determinants of behavioral intention among VR users^39–41^. Shen et al.^42^ utilized the unified theory of acceptance and use of technology (UTAUT) to delineate behaviors associated with VR learning. Their empirical results reveal that behavioral intention in VR learning is influenced by factors such as concrete experience, performance expectancy, effort expectancy, social influence, and facilitating conditions. Désiron et al.^43^ examined determinants of performance and behavioral intention of VR users within the realm of infection prevention. They highlighted that both prior effort expectancy and in-training user engagement play significant roles in influencing performance. Shin^44^ furnished evidence asserting that enhanced usability aids learnability by fostering increased empathy and embodiment within VR learning environments. Like other hedonic Information Systems, VR devices cater to users' entertainment needs^45,46^. Kang et al.^47^ analyzed the media content of BTS, a Korean pop boy band, establishing six distinct groups such as VR video with a headset, news articles, control, among others. The researchers determined a pronounced discrepancy in engagement between the VR headset and news article groups, particularly in the context of immersion in BTS’s content. Al-Sharafi et al.^48^ employed a hybrid SEM-ANN methodology to assess the influence of psychological, social, and quality parameters on the continuous intention to use VR meeting platforms. They confirmed that these factors considerably influence users’ continued intent to engage with virtual meeting platforms. Emphasizing the element of entertainment, Hartmann^45^ posited that it remains paramount in VR experiences. The author suggested that VR allows users to experience pleasurable expansions of self, even outside narrative contexts. Consequently, factors such as perceived usefulness and enjoyment play pivotal roles in shaping users' attitudes and intentions to continue using VR.

### Affordance

Affordance, as a concept, originated from the cognitive theory put forth by Gibson^49^. It is conceptualized as an idea prompting specific actions^49^ and highlights the potential behaviors an object can incite in an animal or human. Given that VR users' actions are predominantly guided by on-screen prompts, the concept of affordance seamlessly integrates into the VR realm. Its applications have spanned areas including human–machine interaction and design. Gaver^50^ postulated that affordances persist irrespective of human awareness. Kaptelinin and Nardi^51^ presented an alternate perspective to^52^’s ecological psychology, portraying technological affordances as opportunities for culturally influenced behaviors. This involves an intricate triad: the individual, mediation instruments, and contextual elements. When users navigate virtual realms via VR devices, it’s these affordances that instigate their actions. Norman^10^ identified affordance design as instrumental in shaping human-object interactions. Hartson^53^ categorized affordances for interaction design into cognitive, sensory, functional, and physical. According to Gross et al.^54^, an affordance-based framework aptly defines experiential reality, especially when designing virtual environments.

The subsequent years witnessed scholars leveraging the concept of affordance to expound on human behaviors, backed by empirical studies. Tsai and Ho^55^, for instance, pinpointed diversity and intuitiveness as key affordance-driven determinants shaping attitudes towards smartphone use. Their findings showcased how diversity influences attitudes through perceived usefulness and ease of use. Given the parallels between VR headsets and smartphones in terms of design and display, it becomes pertinent to delve into VR’s affordances. Categorized immersion and presence as affective affordances, while empathy and embodiment were regarded as educational affordances in VR learning contexts^44^. The study discerned that enhancing affective affordance amplifies usability, fostering educational affordance, and consequently, augmenting learnability. Later, Shin^56^ reframed immersion and presence as virtual affordance and empathy and embodiment as affective affordance for augmented reality games. Here, technology affordance was found to elevate usability, thus facilitating educational affordance and enhancing playability.

Moreover, affordance exploration isn't limited to VR. Research has delved into its implications in internet banking^57^, gaming^58^, the metaverse^59^, mobile applications^14^, and IoT^13^. Consequently, this study taps into such insights to shed light on how affordances influence continuance intention in VR contexts.

### Aesthetics

Aesthetics, a key factor in the realm of marketing, captivates consumers and serves as a distinguishing feature amongst brands^60–62^. Researchers, such as Wiecek et al.^63^, emphasize that aesthetics greatly mold customer preferences during product selection. Jiang et al.^64^ argue for the role of aesthetics in fostering continuance intention among virtual shoppers via attitude. Homburg et al.^65^ through a survey of U.S. consumers, accentuated the capability of aesthetics in amplifying purchase intentions and facilitating positive word-of-mouth (WOM). IT research has also endorsed aesthetics' centrality in charting user behavior patterns. For instance, Toufani et al.^33^ acknowledged aesthetics, encompassing facets like color, design, and shape, as a significant determinant of purchase intentions through emotional and social value within the smartphone domain. Sabir^62^ contends that aesthetics play a dual role in enhancing smartphone user satisfaction.

A pivotal aesthetic component of VR is the headset design. Its size, shape, and weight directly influence user comfort and immersion. Kim et al.^66^ highlighted user preference for headsets that are lightweight and adaptable. Additionally, Katz and Sugiyama^67^ showed that modern, sleek headset designs enhance user perceptions of technology sophistication. Parallelly, the visual aesthetics of VR environments critically shape user experiences. The graphic fidelity and meticulousness impact immersion levels, as evidenced by Slater et al.^68^, who noted a preference for realism. Furthermore, haptic feedback—vibrations or force feedback—intensifies immersion. Lécuyer^69^ showcased user preference for synchronized, authentic haptic feedback. The VR environment's color and layout also influence experiences, with Tussyadiah et al.^70^ noting the profound impact of color on user emotions.

Several researches underscore aesthetics' significance in marketing^71,72^ and IT^30,73^. With this backdrop, our research endeavors to delve into VR aesthetics, aiming to glean insights about consistent VR utilization. In essence, aesthetic considerations in VR gear, be it headset design or visual environments, emerge as paramount in sculpting user experiences, attitudes, and continuance intentions.

### Ethical approval

This study was approved by an institutional review board of HJ Institute of Technology and Management. All methods were performed in accordance with the relevant guidelines and regulations. This research was performed in accordance with the Declaration of Helsinki.

### Informed consent

Informed consent was obtained from all individual participants included in the study.

## Research model

**Figure 1:**
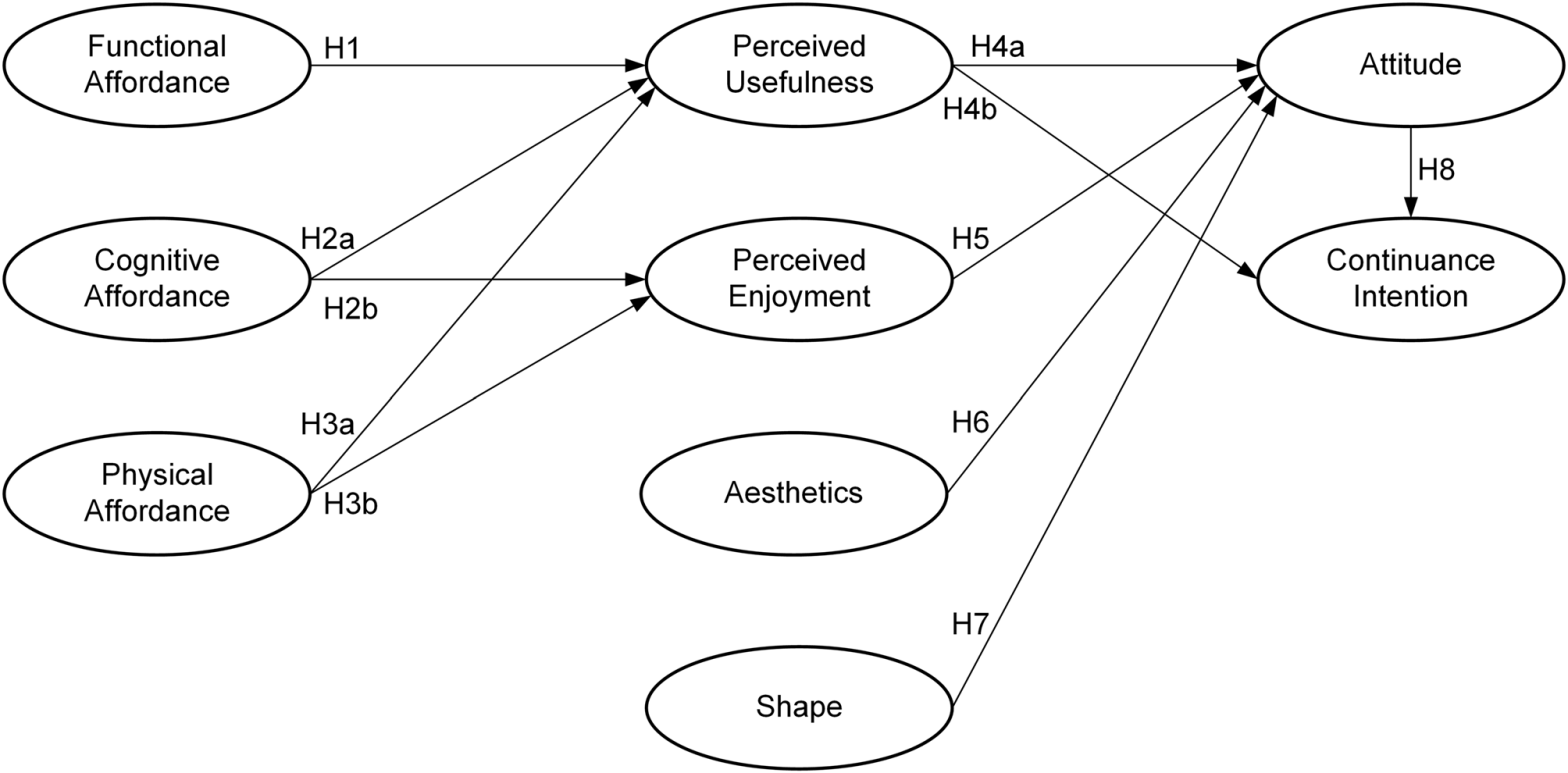
Research model.

Figure 1 presents a research model of this research. This study posits that functional affordance influences perceived usefulness. It suggests that cognitive affordance and physical affordance are the primary antecedents of both perceived usefulness and perceived enjoyment. The research further indicates that attitude is molded by perceived usefulness, perceived enjoyment, aesthetics, and shape. Finally, it proposes that continuance intention is driven by attitude and perceived usefulness.

### Functional affordance

Functional affordance is described as a design element that aids users in performing tasks (i.e., the usefulness of a system function)^53^. It gives users high usefulness and fulfills the purpose of user behaviors^53^. Within the context of technological interfaces, functional affordance directly correlates with users’ perceptions regarding the utility of a specific platform or tool. According to^74^, perceived usefulness is a significant predictor of technology acceptance, and it is largely influenced by the functionality and capabilities of the tool in question. Moreover, when users identify high levels of functional affordance within a system, they often believe that using the system will enhance their task performance^75^. Specifically, in the realm of VR, when VR platforms provide clear functional affordances, users tend to perceive them as more useful for their intended tasks^76,77^. Functional affordances have a significant and positive influence on user satisfaction and UX in the domain of VR^78^. The logical extension of these findings is that an increased sense of functional affordance would positively influence perceptions of usefulness. Hence, this study posits the following hypothesis.

H1: Functional affordance has a positive impact on perceived usefulness.

### Cognitive affordance

Cognitive affordance refers to those aspects of an interface or system design that facilitate mental processes and understanding, guiding users in their interactions^53^. The clarity, intuitiveness, and informational support offered by an interface can help users think, reason, and understand the content or the tasks they are involved in. Within the context of technological adoption, perceived usefulness is one of the foundational determinants. Cognitive affordance, by simplifying complex tasks and enhancing user understanding, can positively influence this perception. When users find an interface cognitively supportive, they are more likely to see it as beneficial for their intended purposes^79^. On a similar note, the enjoyment derived from using a technology is influenced by the ease of understanding and seamless interactions. Systems with higher cognitive affordance tend to reduce cognitive load and frustration, thereby increasing the pleasure and enjoyment of the user experience^80^. Hence, a robust cognitive structure and guidance can enhance both the perceived usefulness and enjoyment for users. Thus, this study suggests the following hypotheses.

H2a: Cognitive affordance has a positive impact on perceived usefulness.
H2b: Cognitive affordance has a positive impact on perceived enjoyment.

### Physical affordance

Physical affordance refers to those design features of an interface or system that amplify or simplify physical interactions^53^. The effectiveness of such design elements is often gauged through their ability to enhance interface navigation, user-friendly layout, and intuitive iconography, especially in IT devices^14^. As posited by^13^, mobile application interfaces frequently integrate physical affordances, aiming to stimulate user engagement and actions. Within the realm of VR, the significance of physical affordance becomes even more pronounced. Interfaces that encompass pronounced and responsive menus, buttons, or touchpoints facilitate more seamless user interaction^78^. Such tangible and responsive design elements not only bolster the ease of interaction but also elevate the user's perception of the system's utility. Moreover, when users encounter a physically intuitive VR environment, the level of enjoyment and immersion they experience tends to heighten^81^. Drawing on these perspectives, when users engage with interfaces that offer favorable physical affordances, they are likely to derive both utility and pleasure. Thus, this study suggests the following hypotheses.

H3a: Physical affordance has a positive impact on perceived usefulness.
H3b: Physical affordance has a positive impact on perceived enjoyment.

### Perceived usefulness

Perceived usefulness is articulated as an individual’s firm belief in the utility offered by a particular technology^16^. This belief acts as a cornerstone for technology adoption and usage across diverse settings, as confirmed by extensive literature^82–85^. Apart from acting as a precursor for technology uptake, perceived usefulness is instrumental in molding user attitudes^20^ and fortifying the intention to continue using the technology^86,87^. Within the specialized domain of VR, performance expectancy, which closely aligns with perceived usefulness, has been recognized as a significant determinant influencing user intentions^42^. Further studies on VR environments have reiterated the impact of perceived usefulness on user behavioral intentions^88,89^. In scenarios where VR extends meaningful and pragmatic support, users tend to harbor favorable impressions and exhibit sustained usage. Drawing from these insights, the present research contends that perceived usefulness will cast a positive effect on both attitude and the intent to persist with VR technology. Thus, this study suggests the following hypotheses.

H4a: Perceived usefulness has a positive impact on attitude.
H4b: Perceived usefulness has a positive impact on continuance intention.

### Perceived enjoyment

Perceived enjoyment encapsulates the pleasure derived from employing a specific IT^75^. This intrinsic pleasure, apart from being a source of hedonic value, manifests as a driving force for individuals’ behavioral intentions^82,90–92^. The continuance intention, or the sustained desire to use a technology, is also propelled by this perception of enjoyment^93^. Moreover, when examining attitudes towards IT tools and systems, it becomes evident that enjoyment plays a crucial role in shaping positive perceptions^24,94,95^. Within the realm of VR, the sentiment remains consistent. Users who derive pleasure from their VR experiences invariably cultivate a favorable outlook towards the technology, as illuminated by research conducted by Jiang et al.^64^. Thus, this study suggests the following hypotheses.

H5: Perceived enjoyment has a positive impact on attitude.

### Aesthetics

Aesthetics, characterized by elements of harmony, order, and beauty in tangible realms, is articulated by^96^. This element is not merely decorative but stands as a decisive factor when consumers make product choices, a sentiment echoed by research from^97^ and^98^. Notably, the visual appeal or screen aesthetics has the potency to elevate user satisfaction, courtesy of the pleasure derived therein, as evidenced by the findings of Liu et al.^99^. Delving into attitudes formed towards electronic gadgets, it becomes clear that aesthetics is instrumental, a perspective reinforced by Hsiao and Chen^98^. Within the expansive realm of VR, a sophisticated aesthetic design plays an integral role. As research by Jiang et al.^64^ postulates, a heightened aesthetic perception within VR environments can boost positive attitudes. The immersive and vivid nature of VR, when accentuated with splendid color palettes and designs, can significantly sway users’ perceptions. Thus, this study suggests the following hypotheses.

H6: Aesthetics has a positive impact on attitude.

### Shape

The shape of a device, especially in the context of VR, is not merely a matter of physical configuration; it serves as a reflection of ergonomic considerations, design principles, and user aesthetics, profoundly influencing the user's interaction with the device. White^96^ highlights the broader understanding of aesthetics, indicating that harmonious designs can elicit positive reactions. Further delving into product design, Bigoin-Gagnan and Lacoste-Badie^97^ underscore the primacy of design elements, including shape, in driving product selection among consumers. The relationship between device shape and users' attitudes is also given credence by Hsiao and Chen^98^, who establish that pleasing design characteristics foster more favorable attitudes towards electronic devices. In the realm of VR, the physical form of the device can significantly shape users' experiences, as postulated by Jiang et al.^64^. A VR device with a shape that resonates with user preferences might bolster their affinity for it, thus potentially enhancing their overall attitude. Thus, this study suggests the following hypotheses.

H7: Shape has a positive impact on attitude.

### Attitude

Attitude is articulated as a person’s evaluative judgement concerning a particular occurrence or entity, a definition rooted in the work of Ajzen^100^. This personal stance, as depicted by numerous research, endeavors plays a pivotal role in molding behavioral intention^95,101–103^. Taking a closer glimpse into the VR realm, attitude emerges as an influential determinant in shaping continuance intention. This correlation is fortified by studies such as those conducted by^64^, Lin^104^ and Qin et al.^105^. Users who harbor a more pronounced positive attitude towards VR are more inclined to consistently engage with the technology, underlining its impact on long-term utilization. Thus, this study suggests the following hypotheses.

H8: Attitude has a positive impact on continuance intention.

## Research methodology

### Measurements

To the extent possible, this study adapted constructs from measurement scales used in prior studies to fit the VR case. The questionnaire was first written by the authors. A Korean professional who is fluent in English translated a questionnaire in English into Korean. The survey results were translated back into English. The two English versions of the questionnaire had only slight differences that were adjusted by the author. Before performing the main survey, the questionnaire indicators were confirmed by three researchers in the IS and marketing fields. All constructs except for demographic information and frequency were gauged using a 7-point Likert scale with suitable ranges. Table A1 shows all the measurement items for the constructs.

### Sample

An online survey was employed to gather the data for the current work. The survey approach enables the generalizability of results, replication of findings, and concurrent evaluation of various elements^106^. This research employed a purposive sampling technique to select the study participants. Purposive sampling, also known as judgmental, selective, or subjective sampling, is a non-probability sampling method that is characterized by the use of discretion in selecting the participants who are most able to contribute valuable data^107^. In this context, our target population consisted of individuals with prior experience using VR, which justified our use of purposive sampling. Before the main survey, a pilot test was conducted to ensure the reliability and validity of the research instrument. A group of 15 participants, who fit the demographic of our target population, was selected for the pilot test. The feedback from the pilot test was used to refine the survey instrument, enhancing its reliability and validity^108^. The online link to the questionnaire was delivered to a diverse population including office workers, university students, and researchers. Participation in the survey was voluntary. After removing incomplete responses, the remaining 240 responses were used for the data analysis.

Table 1 presents demographic data from 240 study respondents. Participants were mostly male (55.8%, n = 134) with a substantial female minority (44.2%, n = 106). Respondents were largely in their 30s (48.3%, n = 116), followed by those in their 20s (26.7%, n = 64) and 40s (16.7%, n = 40). The 10s and 50s age groups were the smallest, comprising only 5% (n = 12) and 3.3% (n = 8) respectively. The VR devices used varied: Oculus was the most popular (44.6%, n = 107), followed by SONY (34.2%, n = 82), HTC (12.5%, n = 30), and DPVR (2.5%, n = 6). Respondents using other VR models comprised 6.3% (n = 15) of the total.

**Table 1:** Profile of the respondents.

Demographics	Item	Subjects (N = 240)	
		Frequency	Percentage
Gender	Male	134	55.8%
	Female	106	44.2%
Age	10s	12	5.0%
	20s	64	26.7%
	30s	116	48.3%
	40s	40	16.7%
	50s	8	3.3%
Model	Oculus	107	44.6%
	SONY	82	34.2%
	HTC	30	12.5%
	DPVR	6	2.5%
	Other	15	6.3%

## Results

The present study analyzed the theoretical framework using the partial least squares structural equation modeling (PLS-SEM) method with SmartPLS 3^109^. The method is adequate for complex research models with a lot of constructs^110^. Additionally, the PLS is advised for prediction frameworks that concentrate on clarifying the major precursors^111^.

### Common method bias

Potential bias arising from common methods was a concern in this study, given that data were collected using a single method and at a single point in time. Firstly, the variance inflation factor (VIF) was calculated for each item, with the maximum value found to be 6.027, comfortably below the threshold of 10, indicating no multicollinearity issues^112^. Second, as a supplementary analysis, we conducted the marker variable procedure^113^. Given the lack of multicollinearity and results of the marker variable procedure, the risk of common method bias seems minimal in this study.

### Measurement model

Cronbach’s alpha and composite reliability (CR) were used to assess the reliability of the model. Both the results for Cronbach’s alpha and CR exceeded 0.7, demonstrating the model's reliability^114^. Convergent validity was assessed using item loadings and the average variance extracted (AVE). All factor loadings were significant and exceeded 0.7. The AVE surpassed the recommended threshold of 0.5, establishing convergent validity^115^. Table 2 displays the test results for reliability and validity.

**Table 2:** Test results of reliability and validity.

Construct	Items	Mean	St. Dev.	Factor loading	Cronbach’s alpha	CR	AVE
Functional affordance	FAF1	4.638	1.251	0.915	0.792	0.906	0.828
	FAF2	4.833	1.383	0.905			
Cognitive affordance	CAF1	4.746	1.136	0.940	0.798	0.906	0.828
	CAF2	4.571	0.933	0.879			
Physical affordance	PAF1	4.975	1.378	0.898	0.900	0.938	0.834
	PAF2	4.554	1.642	0.874			
	PAF3	4.696	1.116	0.966			
Perceived usefulness	PUS1	4.896	1.089	0.947	0.790	0.878	0.708
	PUS2	4.150	1.592	0.811			
	PUS3	4.404	1.772	0.755			
Perceived enjoyment	PEN1	5.600	1.224	0.883	0.908	0.942	0.845
	PEN2	5.163	1.269	0.935			
	PEN3	5.158	1.345	0.938			
Aesthetics	ATH1	5.063	1.313	0.922	0.848	0.907	0.765
	ATH2	4.771	1.061	0.884			
	ATH3	4.792	1.147	0.815			
Shape	SHA1	4.758	1.408	0.935	0.909	0.942	0.844
	SHA2	4.704	1.438	0.894			
	SHA3	4.463	1.533	0.926			
Attitude	ATT1	4.850	1.149	0.934	0.918	0.948	0.860
	ATT2	4.846	1.264	0.895			
	ATT3	4.938	0.780	0.952			
Continuance Intention	COI1	4.725	1.169	0.934	0.759	0.863	0.680
	COI2	4.600	1.396	0.797			
	COI3	4.892	0.920	0.730			

Discriminant validity, which determines the extent to which a construct is truly distinct from other constructs, was evaluated in this study through the Fornell and Larcker criterion^116^ and the Heterotrait-Monotrait ratio (HTMT)^117^. The Fornell and Larcker criterion suggests that the square root of the AVE for each construct should be greater than the correlation shared with any other construct. As shown in Table 3, the square root of the AVE (diagonal values) for each construct is larger than its highest correlation with any other construct, thereby confirming discriminant validity.

**Table 3:** Fornell & Larcker test.

Constructs	1	2	3	4	5	6	7	8	9
1. Functional affordance	0.910								
2. Cognitive affordance	0.663	0.910							
3. Physical affordance	0.535	0.822	0.914						
4. Perceived usefulness	0.638	0.854	0.758	0.841					
5. Perceived enjoyment	0.416	0.588	0.761	0.449	0.919				
6. Aesthetics	0.820	0.815	0.809	0.837	0.593	0.875			
7. Shape	0.702	0.849	0.757	0.743	0.646	0.792	0.919		
8. Attitude	0.490	0.731	0.758	0.673	0.706	0.684	0.757	0.927	
9. Continuance intention	0.472	0.811	0.715	0.733	0.508	0.748	0.804	0.708	0.825

Note: Diagonal elements are the square root of AVE.

On the other hand, the HTMT values are preferably less than 0.90^117^. However, as displayed in Table 4, a few HTMT values exceed the threshold of 0.90. The HTMT criterion is more conservative than the Fornell & Larcker criterion. Thus, exceeding the threshold in HTMT does not immediately imply a serious discriminant validity problem, particularly when Fornell & Larcker criterion and factor loadings suggest adequate discriminant validity^118^. Additionally, HTMT and Fornell & Larcker criterion are supplementary and should not be used as a standalone criterion^117^. Given that the Fornell & Larcker criterion confirmed discriminant validity and the loading of items on their intended constructs was satisfactory, we proceeded with the testing of the structural model.

**Table 4:** HTMT.

Constructs	1	2	3	4	5	6	7	8	9
1. Functional affordance									
2. Cognitive affordance	0.848								
3. Physical affordance	0.626	0.932							
4. Perceived usefulness	0.832	1.069	0.886						
5. Perceived enjoyment	0.492	0.647	0.841	0.528					
6. Aesthetics	1.027	0.974	0.894	1.032	0.649				
7. Shape	0.841	0.982	0.826	0.880	0.696	0.916			
8. Attitude	0.578	0.809	0.832	0.767	0.773	0.761	0.813		
9. Continuance intention	0.615	1.027	0.863	0.930	0.629	0.922	0.967	0.851	

Following the guidelines proposed by Henseler et al.^117^, we assessed the fit through SRMR, dULS, and dG values. The standardized root mean residual (SRMR) values for the saturated and estimated models were 0.123 and 0.129, respectively. The values of dULS and dG, 4.900 and 3.842 for the saturated model, and 5.398 and 4.099 for the estimated model. Furthermore, the normed fit index (NFI) was 0.560 for the saturated model and 0.537 for the estimated model. The chi-square difference was 4045.533 for the saturated model and 4260.839 for the estimated model, further confirming the model fit^117^.

### Structural model

**Figure 2:**
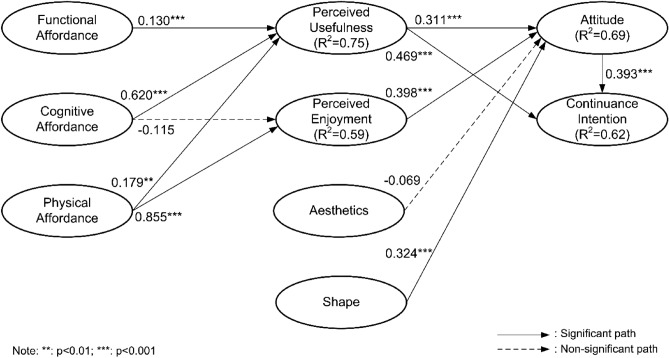
Analysis results (PLS Algorithm).

SEM analysis was conducted to assess the structural model. The current research conducted the Bias-corrected and Accelerated (Bca) bootstrap approach (5000 resamples). As shown in Fig. 2, nine of the eleven paths in the research model are supported.

This research investigated adjusted R^2^, which means the variance described by the model that defines the quality of the overall model^115^. Henseler and Sarstedt^119^ regard R^2^ values of 0.67, 0.33, and 0.19 as substantial, moderate, and weak, each. In our model, the R^2^ scores for endogenous constructs were as follows: Perceived usefulness (0.75, substantial), perceived enjoyment (0.59, moderate), usage (0.69, attitude), and continuance intention (0.62, substantial).

Table 5 details the results of the structural model, elucidating the relationships between various constructs and their respective hypotheses. Hypothesis H1, positing the influence of Functional Affordance on Perceived Usefulness, is supported. H2a, suggesting Cognitive affordance's effect on perceived usefulness, is also supported, whereas H2b, asserting the effect of Cognitive affordance on perceived enjoyment, is not supported. H3a and H3b, indicating the influence of Physical affordance on perceived usefulness and perceived enjoyment, respectively, are both supported. The table further confirms the validity of H4a and H4b, showing significant associations between Perceived usefulness and both attitude and continuance Intention. H5, linking perceived enjoyment to Attitude, is supported, while H6, suggesting an influence of aesthetics on attitude, is not supported. Hypothesis H7, postulating an impact of Shape on Attitude, is supported. Finally, H8, detailing the relationship between attitude and continuance intention, is supported.

**Table 5:** Results of structural model.

H	Cause	Effect	Coefficient	T-value	P-value	Hypothesis
H1	Functional affordance	Perceived usefulness	0.130***	3.344	0.0008	Supported
H2a	Cognitive affordance	Perceived usefulness	0.620***	8.014	0.0000	Supported
H2b	Cognitive affordance	Perceived enjoyment	-0.115	1.253	0.2102	Not supported
H3a	Physical affordance	Perceived usefulness	0.179**	2.725	0.0065	Supported
H3b	Physical affordance	Perceived enjoyment	0.855***	12.155	0.0000	Supported
H4a	Perceived usefulness	Attitude	0.311***	4.281	0.0000	Supported
H4b	Perceived usefulness	Continuance intention	0.469***	8.104	0.0000	Supported
H5	Perceived enjoyment	Attitude	0.398***	9.035	0.0000	Supported
H6	Aesthetics	Attitude	-0.069	0.865	0.3870	Not Supported
H7	Shape	Attitude	0.324***	4.345	0.0000	Supported
H8	Attitude	Continuance intention	0.393***	7.365	0.0000	Supported

Note: For P-value in the table, **: p < 0.01, ***: p < 0.001. For values that are not statistically significant (p > 0.05), the exact p-value is shown.

## Discussion

In our study, we aimed to explore the correlation between key elements of VR, including affordance, usefulness, enjoyment, aesthetics, and shape, and users’ continuance intention. The analysis yielded several significant findings, which have important theoretical and practical implications.

A significant relationship emerged between the functional affordance of VR and perceived usefulness. This implies that when users perceive VR tools as having the capability to provide them with the means to accomplish certain tasks efficiently, they are more likely to find these tools useful. This aligns with prior studies that emphasized the importance of functionality in technology acceptance^76,77^. In the context of VR, this suggests that for a user to embrace this technology, the VR system needs to offer more than just immersive experiences; it should provide functional value that aids users in achieving their objectives.

The significant relationship identified between cognitive affordance and perceived usefulness suggests that when VR tools or other interfaces are designed to align with the user’s cognitive processes, these tools are more likely to be deemed useful. This means that systems that align well with human cognition facilitate understanding, reduce cognitive load, and subsequently enhance the perception of the tool's utility. These findings resonate with established theories such as the cognitive load theory, which emphasizes the importance of managing cognitive processes to enhance learning and task performance^120^. In practical terms, designers and developers should prioritize creating interfaces that naturally fit users’ cognitive patterns, making the technology more intuitive and thereby more useful. However, our findings also indicate a non-significant relationship between cognitive affordance and perceived enjoyment. This suggests that while aligning a tool with cognitive processes can make it useful, it doesn’t necessarily translate into it being enjoyable or entertaining. This is a crucial distinction, especially in contexts where the primary objective is user engagement or entertainment. It implies that merely making a tool cognitively coherent might not suffice in making it pleasurable. This can be attributed to the multifaceted nature of enjoyment, which can be influenced by various factors ranging from aesthetics, novelty, interactive feedback, or personal interests. As highlighted in prior work, perceived enjoyment often transcends utility and can be driven by hedonic, emotional, and sensory aspects^27^.

The results of this study underscore the significant relationship between physical affordance and perceived usefulness. When a VR tool or system integrates physical affordances effectively, it naturally aligns with the user's instincts and expectations, making it easier to navigate, understand, and extract value. This intuitive design enhances the perception of the tool's utility, as users can seamlessly integrate it into their tasks without unnecessary cognitive disruptions. This revelation is consistent with prior studies, which have stressed the vital role of user-friendly designs in determining the perceived value of a digital tool^13,50^. Therefore, designers and developers should emphasize optimizing the physical affordance in VR tools to ensure maximum user adaptability and perceived usefulness. Simultaneously, our findings suggest a significant relationship between physical affordance and perceived enjoyment. This is indicative of the broader implications of physical design elements beyond mere functionality. An interface or system that boasts superior physical affordances is not just easier to use; it also makes the experience more engaging, delightful, and enjoyable. The tactile and physical interactions, when designed well, can stimulate a sense of immersion, playfulness, and satisfaction, crucial for the overall user experience. Such insights echo the sentiments of prior research that underscores the role of holistic design in determining both the utility and pleasure derived from interactive tools^81,121^.

Our study has revealed a significant correlation between perceived usefulness and attitude towards the technology in question. This outcome substantiates the foundational belief that when users discern a technology as beneficial and conducive to their needs, they are more likely to develop a positive attitude towards it. The theoretical underpinning of this result can be traced back to the TAM^16^, which posits perceived usefulness as a primary predictor of user attitude. Essentially, the more users perceive a technology as adding value or making their tasks easier, the more favorably they view it. This provides actionable insight for designers and developers: to foster positive attitudes, one must prioritize the creation and communication of tangible benefits and efficiencies in the technological solutions they offer. Additionally, our analysis also showcases the role of perceived usefulness in influencing continuance intention. Once users recognize the value of a tool (its perceived usefulness), they are not only likely to adopt it but also more inclined to continue using it in the long run. This finding aligns with previous research that emphasized the role of perceived benefits in ensuring sustained user engagement and loyalty^92,122,123^. In essence, perceived usefulness doesn’t just affect initial adoption—it has ramifications on the lifecycle of user interaction with the technology.

Our findings confirm that perceived enjoyment significantly influences users’ attitudes. This suggests that beyond the instrumental benefits of a technology, the experiential and hedonic qualities it offers play a pivotal role in shaping users' overall impressions. This result is consistent with previous research that underscores the role of enjoyment as an intrinsic motivation in using technology^24,82,90–92^. When users find an interface or a platform pleasurable and fun, they are more likely to develop a positive attitude towards it, regardless of its practical functionalities. This association between perceived enjoyment and attitude also resonates with the broader consumer behavior literature, where positive emotional experiences with a product or service often translate into favorable attitudes and, consequently, increased likelihood of repeated use or purchase^124^. In the context of VR, which is inherently interactive and immersive, this link becomes even more pronounced. The sensory-rich experiences that VR platforms offer can amplify the pleasure derived from usage, and as our results indicate, this enjoyment is a strong predictor of positive attitudes. For developers and designers in the VR space, this underscores the importance of integrating elements that enhance enjoyment. While functionality and utility are undeniably vital, they should be complemented with features that elevate the user's hedonic experience.

Contrary to expectations and conventional wisdom that suggests that aesthetics play a significant role in influencing users' attitudes towards technology, our study indicates no significant relationship between the two. This divergence from the established understanding might be attributed to several reasons. One plausible explanation could be the evolving definition of aesthetics in the VR realm. Unlike traditional user interfaces where aesthetics might be equated with visual appeal, in VR, the immersive experience might overshadow the aesthetic attributes. Users might prioritize immersive quality, interactivity, or realism over visual aesthetics per se^125^. Another perspective might be the subjective nature of aesthetics. What is deemed aesthetically pleasing to one user might not resonate with another, making it a less consistent determinant of attitude compared to more objective measures. On the other hand, the influence of the shape of the VR device on attitude is pronounced. This finding emphasizes the tangible aspects of VR experiences. The shape, as a tangible attribute, has direct implications on the user’s comfort, usability, and overall interaction experience. A well-designed VR device, in terms of its shape, can enhance the ergonomic experience, leading to prolonged usage and positive attitudes^126^. This suggests that while users might have evolving preferences for visual elements, the physical comfort and ease of use remain paramount.

Our findings underscore the significant influence of user attitudes on their continuance intention in the context of VR. Historically, the theory of reasoned action (TRA) and its successor, the theory of planned behavior (TPB), have posited that an individual’s attitude towards a behavior directly impacts their intention to perform that behavior^127,128^. Drawing parallels from these theories, our study suggests that when users have a positive attitude towards a VR system, they are more likely to continue using it in the future. This is intuitive; a positive attitude often stems from favorable experiences, perceived usefulness, and overall satisfaction derived from the technology. There are several layers to this relationship. A favorable attitude might be shaped by ease of use, immersive experience, perceived benefits, and the absence of technical glitches or discomfort while using VR. When users have such affirmative experiences, they develop a positive disposition towards the system, making them more inclined to continue using it^74^. However, it’s worth noting that while attitude is a strong predictor, it's not the sole determinant of continuance intention. Other external factors such as alternative technologies, social influence, or changes in personal circumstances can also influence the decision to continue using VR, even if one has a positive attitude towards it. For practitioners, understanding the pivotal role of attitude can guide strategies to enhance user experiences. Ensuring initial positive interactions with VR, addressing pain points promptly, and continuously innovating to match user expectations can help in cultivating a positive attitude, which, in turn, can lead to sustained usage.

## Conclusion

### Implications for theory

The rapid evolution of VR has constantly reshaped our understanding of its impacts on user behaviors and perceptions. As we dive deeper into the realm of VR, we uncover layers of theoretical insights that challenge pre-existing knowledge. This research endeavors to provide a detailed theoretical contribution, by drawing comparisons with earlier studies and highlighting what this investigation uniquely brings to the table. Our first key contribution lies in distinguishing between cognitive and physical affordances and their implications for VR users. Historical research predominantly homogenized affordances, treating them as a single unit impacting user perceptions^35–37^. However, our study intricately dissects this premise. By addressing this gap, this paper offers a novel perspective on the use of VR devices. In this paper, the effect of affordance in building the continuance intention was verified by focusing on the fact that a significant part of the VR experience is conveyed through the screen. Drawing from the research findings, we can conclude the following in response to our research questions: Functional affordance is a significant driver for perceived usefulness in a VR setting, establishing its foundational role in the user experience. Both cognitive and physical affordances have nuanced roles, where cognitive affordance predominantly influences perceived usefulness, while physical affordance greatly impacts perceived enjoyment. Intricacies in the relationship between perceived usefulness, perceived enjoyment, aesthetics, and shape have been identified, showing that together they form a cohesive framework that shapes a user's attitude towards VR. Importantly, this constructed attitude, when paired with perceived usefulness, significantly determines a user’s continuance intention with VR technology, emphasizing the essential interplay of these factors in influencing sustained VR engagement. Therefore, researchers need to design a classification system for screen composition more efficiently. It is valuable to configure the components of the screen to be more perceptible and physically accessible. Unlike small displays of mobile devices such as smartphones and tablets, VR screens are relatively large that users encounter. It would be meaningful if researchers set the arrangement and size of the components at various levels and then explore the case where the user’s response is optimal. This granular understanding paves the way for a more refined approach to studying affordances, prompting future research to consider the multifaceted nature of VR experiences.

Within the burgeoning domain of VR design, the aesthetic paradigm has long been at the forefront, guiding user attitudes and preferences. Seminal works such as those by^129^, and^130^ have articulated the paramount significance of aesthetics in shaping user perspectives towards technological innovations. However, challenging this entrenched belief, our research offers an alternative viewpoint. We posit that the actual shape of VR devices holds a more potent sway in determining user attitudes than previously emphasized aesthetic considerations. This revelation underscores a pivotal paradigm shift. It compels scholars to recalibrate their theoretical frameworks and urges industry professionals to rethink their design strategies. By emphasizing the form factor, or the shape of the device, our findings hint at the possibility of it outweighing the traditionally vaunted aesthetics. This doesn't merely allude to a visual appeal, but extends to the tactile and ergonomic appeal that a device’s shape can offer.

Lastly, the relationship between user perceptions, attitudes, and intentions has been a cornerstone of behavioral studies for decades. The TRA and the TPB set forth by Ajzen^127^ and furthered by Ajzen^128^ have provided foundational insights into these interconnections. However, the rapid evolution and idiosyncrasies of the VR ecosystem call for a more specialized approach to understanding these relationships. Our investigation refines the existing paradigm, revealing that in the VR context, perceived usefulness extends its influence beyond attitudes. Not only does it directly shape attitudes, but it also sets the stage for continuance intentions^82,87,131^, mediated by those very attitudes. Furthermore, the potency of perceived enjoyment in shaping attitudes is clearly demarcated in our findings. Yet, its direct contribution to continuance intention remains subtle and less profound. This layered understanding amplifies the multifaceted dynamics at play within the VR environment. For scholars, it signals the pressing need to evolve beyond traditional models, crafting theories more attuned to the intricate interplay of perceptions, attitudes, and intentions inherent in cutting-edge technologies like VR.

Conclusively, as the frontier of VR technology advances at a breathtaking pace, it beckons a reimagining of theoretical underpinnings that guide our understanding of it. Our study accentuates the pivotal need to break free from the confines of broad, overarching theories that might have been conceived within the context of older technological paradigms. While such theories offer valuable foundational insights, they might fall short in capturing the nuances and intricacies specific to the contemporary VR landscape. Generalized theoretical assumptions risk oversimplifying the multifaceted interactions and implications that VR presents. The findings from our investigation urge scholars to adopt a more nimble and adaptable theoretical stance, continually calibrating their research frameworks to stay attuned to the ever-evolving intricacies of VR. As we tread further into the realm of VR, it's paramount for academic endeavors to be in tandem with the shifting sands of this technology, ensuring that the research remains both relevant and insightful in the face of VR's transformative journey.

### Implications for practice

The revelations stemming from our research distinctly highlight the differences between cognitive and physical affordances within the VR domain. Specifically, while cognitive affordances carve their niche, it's the physical affordances that manifestly influence both the perceived utility and the pleasure users derive from VR. For service providers and product manufacturers in the VR ecosystem, these insights are paramount. Traditional models that advocate a generalized approach may not harness the full potential of VR's multifaceted environment. For educational VR platforms, the emphasis should tilt towards cognitive affordances. A platform equipped with an intuitive interface, for instance, could drastically augment the perceived usefulness, rendering the learning experience more seamless for students^85^. On the flip side, VR platforms designed for entertainment or gaming should pivot towards enhancing physical affordances. Incorporating tangible features, such as haptic feedback, can elevate the immersive experience, driving user satisfaction and overall enjoyment^132^.

Diving into the intricate facets of VR usability and design, it becomes evident that device shape stands as a significant determinant of user attitudes, perhaps even eclipsing the traditionally emphasized aesthetics. This poses a paradigm shift for managers and developers in the VR space. While the initial lure of a beautifully designed VR headset cannot be denied, longevity in user engagement appears to hinge more on ergonomic factors. A device that sits comfortably on the head, for instance, could translate to prolonged usage and a more immersive experience^133^. This underscores the necessity for managers to guide their development teams differently: the aim should not be solely about creating a visually attractive product, but one that, at its core, meets and exceeds user comfort and usability expectations. As VR technology continues to evolve, so must our approach to design and development, ensuring that user-centric considerations are always at the forefront.

Deep diving into the subtleties of how users perceive and relate to VR technologies, our study paints a detailed picture that holds invaluable lessons for marketing professionals. The salience of perceived enjoyment in shaping attitudes is particularly noteworthy. Rather than spotlighting solely the practical advantages of a VR device, marketers might find it more effective to underscore the sheer enjoyment and immersive experiences their product offers^134^. Imagining an advertisement campaign that vividly brings to life the exhilarating adventures, captivating landscapes, and thrilling narratives possible with a particular VR headset can be potent in influencing prospective buyers. Moreover, while functionality remains crucial, the joy of the experience can be a compelling sell. It's the difference between portraying a VR device as merely a tool and presenting it as a gateway to unparalleled adventures. Furthermore, the ergonomic benefits shouldn’t be sidelined. By juxtaposing the immersive 'fun' experience with the comfort of prolonged use due to superior design, marketers can position their VR offerings as not just devices, but comprehensive experiences that cater to both the heart and the body.

For users navigating the ever-expanding VR marketplace, our research offers a compass to make more informed and satisfactory choices. Recognizing the influence of physical affordances on perceived usefulness and enjoyment empowers them to look beyond mere aesthetics or technical specifications. A device might be packed with the latest features, but if it doesn’t fit comfortably or fails to provide intuitive user interactions, the overall VR experience may fall short of expectations. Moreover, while traditionally, practicality and utility have been paramount in tech purchases, our findings underscore the importance of the 'enjoyment' factor in the realm of VR. This insight is especially significant for users for whom VR isn’t just a tool, but also a means of entertainment or relaxation. They should be seeking out devices or applications that promise not just functionality, but a rich, immersive, and enjoyable experience. It's about finding the right balance between work and play, practicality and pleasure, making their investment in VR truly rewarding.

### Limitation and future research

While our research contributes to the field, it does come with some limitations. Firstly, the respondents surveyed were from a single country, thereby limiting the generalizability of our findings. Cultural or national factors may influence the use of VR devices, which future research could explore by conducting surveys in various countries, thus enhancing the universality of the research model. Secondly, we didn’t adhere to best practices by omitting subscales and item codes in our questionnaire presentation. This oversight could impact data interpretability and challenges future replication efforts. In future research, we aim to meticulously follow these standards for clearer methodology. Thirdly, we acknowledge the difficulties associated with measuring constructs using only two items, which could potentially affect their reliability and validity. The task of identifying comprehensive measures for functional and cognitive affordance within the VR context was particularly challenging. Our item selection aimed to represent these constructs as accurately as possible, but the intricate and multidimensional nature of these constructs may not be entirely captured. We encourage future research to include a broader range of items to enhance the construct's validity and reliability. Lastly, while we verified discriminant validity through the Fornell & Larcker criterion, the HTMT analysis highlighted potential overlap among some constructs. Despite the stringent nature of the HTMT criterion, this issue underscores the need for refining constructs in future research to improve discriminant validity and minimize potential measurement overlap.

### Supplementary Information

Supplementary Information.

## Data Availability

The datasets used and/or analyzed during the current study available from the corresponding author on reasonable request.
