# Supplementary material for: Affordance, usefulness, enjoyment, and aesthetics in sustaining virtual reality engagement
Source: Sci Rep. 2023 Sep 12;13:15097. doi: 10.1038/s41598-023-42113-1 (PMC10497621; doi:10.1038/s41598-023-42113-1)
Supplement: Supplementary file 1 — Supplementary Information. [file 41598_2023_42113_MOESM1_ESM.docx]

# Appendix

Table A1. List of Constructs and Items

| Construct | Items | Mean | Reference |
| --- | --- | --- | --- |
| Functional  Affordance | FAF1 | The VR screen is configured according to the classification system for me. | [Park and Lee^78^](#_ENREF_78) |
|  | FAF2 | The VR screen's menu structure efficiently provides the information I need. |  |
| Cognitive  Affordance | CAF1 | It is easy to understand the function of each button on the VR screen. | [Park and Lee^78^](#_ENREF_78) |
|  | CAF2 | I can comprehend the meaning of all icons on the VR screen. |  |
| Physical  Affordance | PAF1 | The buttons on the VR screen are adequately large. | [Park and Lee^78^](#_ENREF_78) |
|  | PAF2 | The icons on the VR screen are sufficiently large. |  |
|  | PAF3 | The buttons or menu on the VR screen react swiftly. |  |
| Perceived  Usefulness | PUS1 | VR devices are useful. | [Davis^16^](#_ENREF_16) |
|  | PUS2 | VR devices allow me to work more efficiently. |  |
|  | PUS3 | Using VR increases productivity and enhances work (academic) efficiency. |  |
| Perceived  Enjoyment | PEN1 | Experiencing VR is pleasurable. | [Davis^16^](#_ENREF_16) |
|  | PEN2 | Using VR is fun. |  |
|  | PEN3 | Using VR is exciting. |  |
| Aesthetics | ATH1 | The design of the VR screen (e.g., color and menu) is attractive. | [Toufani, et al.^33^](#_ENREF_33) |
|  | ATH2 | The content on the VR screen appears professionally designed. |  |
|  | ATH3 | VR screen components (e.g., icons and compositions) are well-designed. |  |
| Shape | SHA1 | The VR device's shape (e.g., square, oval, and smooth edges) is well-crafted. | [Toufani, et al.^33^](#_ENREF_33) |
|  | SHA2 | The shape of the VR device is appealing. |  |
|  | SHA3 | The shape of the VR device is interesting to look at. |  |
| Attitude | ATT1 | I enjoy using VR devices. | [Davis^16^](#_ENREF_16) |
|  | ATT2 | Using VR devices is a wise decision. |  |
|  | ATT3 | It is reasonable to use VR devices. |  |
| Continuance  Intention | COI1 | I intend to continue using this VR device in the future. | [Bhattacherjee^122^](#_ENREF_122) |
|  | COI2 | I plan to use VR devices continually in the future. |  |
|  | COI3 | I would recommend others to use VR devices. |  |
